# Supplementary material for: Acoustic meta-atom with experimentally verified maximum Willis coupling
Source: Nat Commun. 2019 Jul 17;10:3148. doi: 10.1038/s41467-019-10915-5 (PMC6637156; doi:10.1038/s41467-019-10915-5)
Supplement: Supplementary file 1 — Supplementary Information [file 41467_2019_10915_MOESM1_ESM.pdf]

Acoustic meta-atom with experimentally verified maximum Willis coupling
Supplementary Material

Anton Melnikov,[1,2,3,4,*] Yan Kei Chiang,[4] Li Quan,[5] Sebastian Oberst,[3] Andrea Alù,[6,5] Steffen Marburg,[1] and David Powell[4,†]

[1] *Vibroacoustics of Vehicles and Machines*
Technical University of Munich, Germany

[2] *SBS Bühnentechnik GmbH, Dresden, Germany*

[3] *Centre for Audio, Acoustics and Vibration*
University of Technology Sydney, Australia

[4] *School of Engineering and Information Technology*
University of New South Wales, Canberra, Australia

[5] *Department of Electrical and Computer Engineering*
The University of Texas at Austin, Austin, Texas 78712, USA

[6] *Photonics Initiative, Advanced Science Research Center*
City University of New York, USA

(Dated: June 2, 2019)

SUPPLEMENTARY NOTE 1:

TIME CONVENTION AND INCIDENT FIELD

The incident pressure for a plane wave is given by

$$p_{\mathrm{inc}}(t, \mathbf{x}) = p_0 e^{i(-\omega t + \mathbf{k}\mathbf{x})} \tag{S1}$$

with p_0 as pressure amplitude, $\mathbf{k}$ as wave vector, $\mathbf{x}$ as position vector, ω as angular frequency and t as time.

Considering the linearized and simplified Euler's equation

$$-\rho_0 \frac{d\mathbf{v}}{\mathrm{d}t} = \nabla p \tag{S2}$$

the velocity vector $\mathbf{v}$ can be described as following

$$\mathbf{v}(t, \mathbf{x}) = \frac{1}{i\rho_0 ck} \nabla p(t, \mathbf{x}). \tag{S3}$$

For the velocity field in 2D it can be rearranged to

$$\begin{aligned} \mathbf{v}(t, \mathbf{x}) &= \frac{1}{i\rho_0 ck} \begin{bmatrix} \frac{\partial}{\partial x} \\ \frac{\partial}{\partial y} \end{bmatrix} p_0 e^{i(-\omega t + k_x x + k_y y)} \\ &= \frac{1}{\rho_0 ck} \begin{bmatrix} k_x \\ k_y \end{bmatrix} p_0 e^{i(-\omega t + k_x x + k_y y)} \\ &= \frac{\mathbf{k}}{\rho_0 ck} p_0 e^{i(\omega t - \mathbf{k}\mathbf{x})} = \frac{\mathbf{k}}{\rho_0 ck} p(t, \mathbf{x}) \end{aligned} \tag{S4}$$

with ρ_0 as density and c as speed of sound. For practical reasons numerical differentiation is often required, giving the following approximate expression for the velocity

$$\mathbf{v}(t, \mathbf{x}) \approx \frac{1}{i\rho_0 ck} \frac{\Delta p}{\Delta \mathbf{x}} = \frac{1}{i\rho_0 ck} \frac{p(\mathbf{x}_2) - p(\mathbf{x}_1)}{\mathbf{x}_2 - \mathbf{x}_1} \tag{S5}$$

SUPPLEMENTARY NOTE 2:
POLARIZABILITY EXTRACTION FROM NUMERICAL AND EXPERIMENTAL RESULTS

The incident pressure within a 2D system p_{inc} is represented by a cylindrical function expansion

$$p_{\text{inc}} = \sum_{n=-\infty}^{\infty} \beta_n J_n(kr) e^{in\theta} \tag{S6}$$

and scattered pressure p_{scat} by

$$p_{\text{scat}} = \sum_{n=-\infty}^{\infty} \gamma_n H_n^{(1)}(kr) e^{in\theta} \tag{S7}$$

with β_n as the incident field coefficient, γ_n as the scattered field coefficient, $J_n(kr)$ as Bessel function of order n, $H_n^{(1)}(kr)$ as Hankel function of first kind of order n and r and θ as cylindrical coordinates. If pressure data $p_{\text{scat}}(r,\theta)$ is available from an experiment or numerical simulation, β_n and γ_n coefficients can be obtained from the orthogonality of the exponential functions. To calculate these coefficients we hold radius constant at R^{inc} and R^{scat} respectively

$$\beta_n = \frac{1}{2\pi J_n(kR^{\text{inc}})} \int_0^{2\pi} p_{\text{inc}}(R^{\text{inc}},\theta) e^{-in\theta} \mathrm{d}\theta \tag{S8}$$

and

$$\gamma_n = \frac{1}{2\pi H_n^{(1)}(kR^{\text{scat}})} \int_0^{2\pi} p_{\text{scat}}(R^{\text{scat}},\theta) e^{-in\theta} \mathrm{d}\theta. \tag{S9}$$

The process for choosing R^{inc} and R^{scat} is outlined in Ref. [1].

These integrals are numerically approximated, with the scattered field given by

$$\gamma_n \approx \frac{1}{H_n^{(1)}(kR^{\text{scat}})} \sum_{i=1}^{n} p_{\text{scat}}(\theta_i) e^{-in\theta_i} \frac{1}{n}. \tag{S10}$$

We compare this approach with the acoustic dipole and monopole moments as defined in Ref. [2]

$$M = \int_V \rho dV \tag{S11}$$

$$\mathbf{D} = \int_V \rho \mathbf{r} dV. \tag{S12}$$

We compare the scattered field from a monopole with the first term in the cylindrical function expansion

$$\begin{aligned} p_{Ms} &= \gamma_0 H_0^{(1)}(kr) \\ &= -\frac{ik^2c^2M}{4} H_0^{(1)}(kr) \end{aligned} \tag{S13}$$

resulting in the following expression for the monopole coefficient

$$M = \frac{-4\gamma_0}{ik^2c^2}. \tag{S14}$$

A similar comparison is performed between the dipole scattering and the $n = \pm 1$ terms of the cylindrical function expansion

$$\begin{aligned} p_{Ds} &= (\gamma_1 H_1^{(1)}(kr)e^{i\theta} + \gamma_{-1} H_{-1}^{(1)}(kr)e^{-i\theta}) \\ &= (\gamma_1 e^{i\theta} - \gamma_{-1} e^{-i\theta}) H_1^{(1)}(kr) \\ &= ((\gamma_1 - \gamma_{-1})\cos\theta + (\gamma_1 + \gamma_{-1}) i \sin\theta) H_1^{(1)}(kr) \\ &= -i\frac{k^3c^2}{4}(D_x \cos\theta + D_y \sin\theta) H_1^{(1)}(kr) \end{aligned} \tag{S15}$$

resulting in

$$\begin{aligned} D_x &= -(\gamma_1 - \gamma_{-1})\frac{4}{ik^3c^2} \\ D_y &= -(\gamma_1 + \gamma_{-1})\frac{4}{k^3c^2}. \end{aligned} \tag{S16}$$

This can be summarized by a matrix

$$\begin{bmatrix} M \\ D_x \\ D_y \end{bmatrix} = \frac{-4}{ik^2c^2} \begin{bmatrix} 0 & 1 & 0 \\ \frac{-1}{k} & 0 & \frac{1}{k} \\ \frac{i}{k} & 0 & \frac{i}{k} \end{bmatrix} \begin{bmatrix} \gamma_{-1} \\ \gamma_0 \\ \gamma_1 \end{bmatrix} \tag{S17}$$

connecting the γ coefficients to monopole and dipole moments. The excitation of the monopole and dipole moments by an incident field can be described by a polarizability tensor $\boldsymbol{\alpha}$ in the form [2]

$$\begin{bmatrix} M \\ D_x \\ D_y \end{bmatrix} = \begin{bmatrix} \alpha^{pp} & \alpha_x^{pv} & \alpha_y^{pv} \\ \alpha_x^{vp} & \alpha_{xx}^{vv} & \alpha_{xy}^{vv} \\ \alpha_y^{vp} & \alpha_{yx}^{vv} & \alpha_{yy}^{vv} \end{bmatrix} \begin{bmatrix} \breve{p}^{\text{inc}} \\ \breve{v}_x^{\text{inc}} \\ \breve{v}_y^{\text{inc}} \end{bmatrix}, \tag{S18}$$

where $\breve{p}^{\text{inc}}$ and $\breve{\mathbf{v}}^{\text{inc}}$ represent the incident pressure and velocity at the center of the meta-atom.

When the monopole and dipole moments and the incident pressure are known, we can obtain the polarizability tensor $\boldsymbol{\alpha}$ row by row for M, D_x and D_y from different incident angles $\theta_{1,2,3}$.

$$\begin{bmatrix} \alpha^{pp} & \alpha_x^{pv} & \alpha_y^{pv} \\ \alpha_x^{vp} & \alpha_{xx}^{vv} & \alpha_{xy}^{vv} \\ \alpha_y^{vp} & \alpha_{yx}^{vv} & \alpha_{yy}^{vv} \end{bmatrix} = \underbrace{\begin{bmatrix} \breve{p}^{\mathrm{inc}}(\theta_1) & \breve{v}_x^{\mathrm{inc}}(\theta_1) & \breve{v}_y^{\mathrm{inc}}(\theta_1) \\ \breve{p}^{\mathrm{inc}}(\theta_2) & \breve{v}_x^{\mathrm{inc}}(\theta_2) & \breve{v}_y^{\mathrm{inc}}(\theta_2) \\ \breve{p}^{\mathrm{inc}}(\theta_3) & \breve{v}_x^{\mathrm{inc}}(\theta_3) & \breve{v}_y^{\mathrm{inc}}(\theta_3) \end{bmatrix}^{-1}}_{\boldsymbol{\Upsilon}^{-1}} \begin{bmatrix} M(\theta_1) & D_x(\theta_1) & D_y(\theta_1) \\ M(\theta_2) & D_x(\theta_2) & D_y(\theta_2) \\ M(\theta_3) & D_x(\theta_3) & D_y(\theta_3) \end{bmatrix}. \tag{S19}$$

If data is available for additional incident angles is the polarizability tensor can be found by least squares

$$\boldsymbol{\alpha} = (\boldsymbol{\Upsilon}^T \boldsymbol{\Upsilon})^{-1} \boldsymbol{\Upsilon}^T \begin{bmatrix} M(\theta_1) & D_x(\theta_1) & D_y(\theta_1) \\ \vdots & \vdots & \vdots \\ M(\theta_N) & D_x(\theta_N) & D_y(\theta_N) \end{bmatrix}. \tag{S20}$$

To meaningfully compare the different elements of the polarizability tensor, which have different units, the normalized polarizability tensor $\boldsymbol{\alpha}'$ is introduced as

$$\begin{bmatrix} -\sqrt{2}M \\ ikD_x \\ ikD_y \end{bmatrix} = \begin{bmatrix} -2\alpha^{pp} & \frac{-\sqrt{2}}{\rho c}\alpha_x^{pv} & \frac{-\sqrt{2}}{\rho c}\alpha_y^{pv} \\ ik\sqrt{2}\alpha_x^{vp} & \frac{ik}{\rho c}\alpha_{xx}^{vv} & \frac{ik}{\rho c}\alpha_{xy}^{vv} \\ ik\sqrt{2}\alpha_y^{vp} & \frac{ik}{\rho c}\alpha_{yx}^{vv} & \frac{ik}{\rho c}\alpha_{yy}^{vv} \end{bmatrix} \begin{bmatrix} \frac{1}{\sqrt{2}}\breve{p}^{\mathrm{inc}} \\ \rho c \breve{v}_x^{\mathrm{inc}} \\ \rho c \breve{v}_y^{\mathrm{inc}} \end{bmatrix}, \tag{S21}$$

which satisfies $\boldsymbol{\alpha}' = \boldsymbol{\alpha}'^{T-}$ [2] (i.e. the off-diagonal terms are anti-symmetric).

SUPPLEMENTARY NOTE 3:
HELMHOLTZ-RESONATOR POLARIZABLITY

General Model

Consider a Helmholtz resonator, containing an internal compressible volume V, with multiple incompressible apertures of length l and cross-section A_n. We model this as a 2D system, and set height $h = 1$ for simplicity in all analysis.

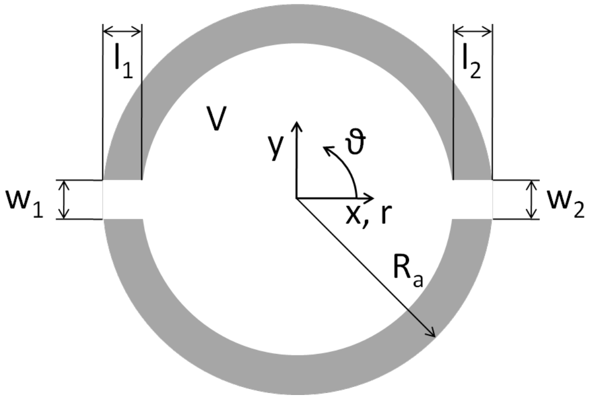

Supplementary Figure 1. Meta-atom geometry.

The air in each of the channels is treated as an incompressible mass of cross-section $A_n = w_n \cdot h$, and when it moves outwards by distance ξ_n, it leads to condensation

$$\Delta\rho/\rho = -\Delta V/V = A_n\xi_n/V \tag{S22}$$

In the acoustic approximation, this condensation leads to an internal pressure increase p^{int}

$$p^{\text{int}} = K\Delta p/p = -KA_n\xi_n/V \tag{S23}$$

where K is the bulk modulus. Considering multiple masses, we arrive at

$$p^{\text{int}} = -\frac{K}{V}\sum_m A_m\xi_m \tag{S24}$$

An incident acoustic field results in some external pressure p^{ext}. In general this is different for each aperture. The inward-directed net force on mass n when subject to external pressure p_n^{ext} is balanced by p^{int} to give

$$F_p = A_n(p_n^{\text{ext}} - p^{\text{int}}) \tag{S25}$$

The movement of the mass in the channels leads to an additional radiation force which must also be included in the balance equation

$$F_n^{\text{rad}} = R_n^{\text{rad}} \frac{\mathrm{d}\xi_n}{\mathrm{d}t} = \frac{\rho_0 c k^2 A_n^2}{8\pi} \frac{\mathrm{d}\xi_n}{\mathrm{d}t} \tag{S26}$$

So the total force on mass n is

$$F_n = A_n(p_n^{\text{ext}} - p^{\text{int}}) + F_n^{\text{rad}} \tag{S27}$$

By Newton's law, this gives the acceleration

$$F_n = -m_n \frac{\mathrm{d}^2\xi_n}{\mathrm{d}t^2} \tag{S28}$$

Given that the mass in each channel is $m_n = \rho_0 A_n l_n$

$$F_n = -\rho_0 A_n l_n \frac{\mathrm{d}^2\xi_n}{\mathrm{d}t^2} \tag{S29}$$

with l_n as the effective length [3]

$$l_n = l + 1.6 \cdot w_n \tag{S30}$$

Substituting the explicit expression for the total force

$$A_n(p_n^{\text{ext}} - p^{\text{int}}) + F_n^{\text{rad}} = -\rho_0 A_n l_n \frac{\mathrm{d}^2\xi_n}{\mathrm{d}t^2} \tag{S31}$$

Factor out the area A_n, and consider $p_n^{\text{rad}} = F_n^{\text{rad}}/A_n$

$$p_n^{\text{ext}} - p^{\text{int}} + p_n^{\text{rad}} = -\rho_0 l_n \mathrm{d}^2\xi_n \mathrm{d}t^2 \tag{S32}$$

An explicit expression for the radiation pressure component can be derived from Kinsler [3, Section 10.8], which gives the radiation force as

$$p_n^{\text{rad}} = \frac{\rho_0 c k^2 A_n}{8\pi} \frac{\mathrm{d}\xi_n}{\mathrm{d}t} = \frac{\rho_0 c}{8\pi} \frac{\omega^2}{c^2} A_n \frac{\mathrm{d}\xi_n}{\mathrm{d}t} = \frac{\rho_0 A_n}{8\pi c} \omega^2 \frac{\mathrm{d}\xi_n}{\mathrm{d}t} \tag{S33}$$

For harmonic waves, note that $\mathrm{d}^2/\mathrm{d}t^2 \to -\omega^2$, so we can write this as

$$p_n^{\text{rad}} = -\frac{\rho_0 A_n}{8\pi c} \frac{\mathrm{d}^3\xi_n}{\mathrm{d}t^3} \tag{S34}$$

Reorder Newton's equation for mass n

$$-\rho_0 l_n \frac{\mathrm{d}^2\xi_n}{\mathrm{d}t^2} + p^{\text{int}} - p_n^{\text{rad}} = p_n^{\text{ext}} \tag{S35}$$

Substituting explicit expressions for p^{int} and p^{rad} leads to

$$\frac{\rho_0 A_n}{8\pi c}\frac{\mathrm{d}^3\xi_n}{\mathrm{d}t^3} - \rho_0 l_n \frac{\mathrm{d}^2\xi_n}{\mathrm{d}t^2} - \frac{K}{V}\sum_m A_m \xi_m = p_n^{\text{ext}} \tag{S36}$$

Using $\exp(-i\omega t)$ time convention leads to

$$i\omega^3 \frac{\rho_0 A_n}{8\pi c}\xi_n + \omega^2 \rho_0 l_n \xi_n - \frac{K}{V}\sum_m A_m \xi_m = p_n^{\text{ext}}. \tag{S37}$$

For a Helmholtz resonator with two apertures we can rewrite Eq. (S37) in matrix notation

$$\underbrace{\left(i\omega^3 \underbrace{\frac{\rho_0}{8\pi c}\begin{bmatrix} A_1 & 0 \\ 0 & A_2 \end{bmatrix}}_{\mathbf{R}} + \omega^2 \underbrace{\rho_0 \begin{bmatrix} l_1 & 0 \\ 0 & l_2 \end{bmatrix}}_{\mathbf{M}} - \underbrace{\frac{K}{V}\begin{bmatrix} A_1 & A_2 \\ A_1 & A_2 \end{bmatrix}}_{\mathbf{K}} \right)}_{\mathbf{K}_{\text{eq}}} \boldsymbol{\xi} = \mathbf{p}^{\text{ext}}. \tag{S38}$$

To obtain the undamped eigenfrequency we neglect the radiative part $\mathbf{R}$ and end up with the eigenvalue problem

$$\det\left(\frac{K}{V}\begin{bmatrix} A_1 & A_2 \\ A_1 & A_2 \end{bmatrix} - \omega^2 \rho_0 \begin{bmatrix} l_1 & 0 \\ 0 & l_2 \end{bmatrix} \right) = 0 \tag{S39}$$

which results in

$$\omega_0 = \sqrt{\frac{c^2}{V}\left(\frac{A_1}{l_1} + \frac{A_2}{l_2}\right)} \tag{S40}$$

and matches the common Helmholtz resonator considerations with two openings [4, 5]. Considering specifically the 2D case with height normalized to 1, the general solution for N apertures is

$$\omega_0 = \frac{c}{a-l}\sqrt{\frac{\sum_{n=1}^{N} w_n}{\pi l}}. \tag{S41}$$

This model yields only one non-zero frequency mode independent of the number of apertures, since it assumes a homogeneous pressure distribution within the internal volume. The full solution accounting for radiative damping can be obtained by inversion as

$$\boldsymbol{\xi} = \mathbf{K}_{\text{eq}}^{-1}\mathbf{p}^{\text{ext}}. \tag{S42}$$

Polarizability Tensor of Helmholtz Resonator with Two Apertures

We perform a multipole expansion considering the fluid displacement ξ_n in every aperture to obtain the monopole moment

$$M = \rho_0 \int_S \xi \mathrm{d}S = \rho_0 \sum_n \xi_n A_n \tag{S43}$$

and dipole moment

$$D = \rho_0 \int_S \xi r \mathrm{d}S = \rho_0 \sum_n \xi_n r_n A_n \tag{S44}$$

resulting in

$$\begin{bmatrix} M \\ D \end{bmatrix} = \rho_0 \begin{bmatrix} A_1 & A_2 \\ x_1 A_1 & x_2 A_2 \end{bmatrix} \boldsymbol{\xi} = \mathbf{A}_{MD}\boldsymbol{\xi}. \tag{S45}$$

which can be inverted to yield

$$\boldsymbol{\xi} = \begin{bmatrix} \frac{x_2}{A_1\rho_0(x_1-x_2)} & \frac{1}{A_1\rho_0(x_1-x_2)} \\ \frac{x_1}{A_2\rho_0(x_1-x_2)} & -\frac{1}{A_2\rho_0(x_1-x_2)} \end{bmatrix} \begin{bmatrix} M \\ D \end{bmatrix} = \mathbf{A}_{MD}^{-1} \begin{bmatrix} M \\ D \end{bmatrix}. \tag{S46}$$

This connects the motion of the effective masses of the Helmholtz resonator to M and D. To find the polarizability of the Helmholtz resonator we must link the external pressure at each aperture p^{ext} to the incident pressure and velocity at the center of the meta-atom, under the assumption that its radius is small compared to the wavelength

$$\breve{p}^{\mathrm{inc}} = \frac{p_1^{\mathrm{ext}} + p_2^{\mathrm{ext}}}{2} \tag{S47}$$

and based on Eq. (S5)

$$\breve{v}_x^{\mathrm{inc}} = \frac{p_2^{\mathrm{ext}} - p_1^{\mathrm{ext}}}{i2a\rho_0\omega} \tag{S48}$$

with $a = R$ as particle radius. We can write it in a matrix form as

$$\begin{bmatrix} \breve{p}^{\mathrm{inc}} \\ \breve{v}_x^{\mathrm{inc}} \end{bmatrix} = \begin{bmatrix} \frac{1}{2} & \frac{1}{2} \\ -\frac{1}{i2a\rho_0\omega} & \frac{1}{i2a\rho_0\omega} \end{bmatrix} \begin{bmatrix} p_1^{\mathrm{ext}} \\ p_2^{\mathrm{ext}} \end{bmatrix} = \mathbf{A}_{pv}\mathbf{p}^{\mathrm{ext}} \tag{S49}$$

or as the inverse

$$\mathbf{p}^{\mathrm{ext}} = \begin{bmatrix} 1 & -ia\rho_0\omega \\ 1 & ia\rho_0\omega \end{bmatrix} \begin{bmatrix} \breve{p}^{\mathrm{inc}} \\ \breve{v}_x^{\mathrm{inc}} \end{bmatrix} = \mathbf{A}_{pv}^{-1} \begin{bmatrix} \breve{p}^{\mathrm{inc}} \\ \breve{v}_x^{\mathrm{inc}} \end{bmatrix} \tag{S50}$$

Now we can combine Eqs. (S45), (S42) and (S50) and write

$$\begin{aligned} \begin{bmatrix} M \\ D_x \end{bmatrix} &= \mathbf{A}_{MD}\boldsymbol{\xi} \\ &= \mathbf{A}_{MD}\mathbf{K}_{\mathrm{eq}}^{-1}\mathbf{p}^{\mathrm{ext}} \\ &= \mathbf{A}_{MD}\mathbf{K}_{\mathrm{eq}}^{-1}\mathbf{A}_{pv}^{-1} \begin{bmatrix} \breve{p}^{\mathrm{inc}} \\ \breve{v}_x^{\mathrm{inc}} \end{bmatrix} \\ \begin{bmatrix} M \\ D_x \end{bmatrix} &= \boldsymbol{\alpha}_{\mathrm{res}} \begin{bmatrix} \breve{p}^{\mathrm{inc}} \\ \breve{v}_x^{\mathrm{inc}} \end{bmatrix} \end{aligned} \tag{S51}$$

which directly gives us the polarizability tensor $\boldsymbol{\alpha}$. The inverse of the polarizability tensor is required later in this derivation and it can be obtained as

$$\begin{aligned}\boldsymbol{\alpha}_{\text{res}}^{-1} &= \mathbf{A}_{pv}\mathbf{K}_{\text{eq}}\mathbf{A}_{MD}^{-1} \\ &= \begin{bmatrix} \frac{1}{2} & \frac{1}{2} \\ -\frac{1}{i2a\rho_0\omega} & \frac{1}{i2a\rho_0\omega} \end{bmatrix} \left(i\omega^3\mathbf{R} + \omega^2\mathbf{M} - \mathbf{K}\right) \begin{bmatrix} \frac{x_2}{A_1\rho_0(x_1-x_2)} & \frac{1}{A_1\rho_0(x_1-x_2)} \\ \frac{x_1}{A_2\rho_0(x_1-x_2)} & -\frac{1}{A_2\rho_0(x_1-x_2)} \end{bmatrix}.\end{aligned} \tag{S52}$$

Polarizability Tensor for Resonator with One Aperture

To obtain the highest possible Willis coupling for the Helmholtz resonator the highest level of asymmetry should be applied by closing one of the two channels. Now the response of the resonator given by Eq. (S37) becomes a scalar equation

$$\left(i\omega^3\frac{\rho_0 A_1}{8\pi c} + \omega^2\rho_0 l_1 - \frac{K}{V}A_1\right)\xi_1 = K_{\text{eq}}\xi_1 = p_1^{\text{ext}}. \tag{S53}$$

This implies further simplifications of Eq. (S51) to

$$\begin{aligned}\begin{bmatrix} M \\ D_x \end{bmatrix} &= \rho_0 \begin{bmatrix} A_1 \\ x_1 A_1 \end{bmatrix} \frac{1}{K_{\text{eq}}} \begin{bmatrix} 1 & -ia\rho_0\omega \end{bmatrix} \begin{bmatrix} \breve{p}^{\text{inc}} \\ \breve{v}_x^{\text{inc}} \end{bmatrix} \\ &= \frac{\rho_0}{i\omega^3\frac{\rho_0 A_1}{8\pi c} + \omega^2\rho_0 l_1 - \frac{K}{V}A_1} \begin{bmatrix} A_1 & -ia\rho_0\omega A_1 \\ x_1 A_1 & -ia\rho_0\omega x_1 A_1 \end{bmatrix} \begin{bmatrix} \breve{p}^{\text{inc}} \\ \breve{v}_x^{\text{inc}} \end{bmatrix} \\ &= \boldsymbol{\alpha}_{\text{res}} \begin{bmatrix} \breve{p}^{\text{inc}} \\ \breve{v}_x^{\text{inc}} \end{bmatrix}\end{aligned} \tag{S54}$$

The polarizability tensor is now a results of an outer product of two vectors, which gives a zero determinant. The tensor is not invertible in that case, so it will be treated differently in the coupling procedure outlined below.

SUPPLEMENTARY NOTE 4:
CYLINDER POLARIZABILITY

The meta-atom consists of a resonator inside a cylindrical body. The direct scattering from the outer cylinder surface contributes to the total scattering of the whole meta-atom and should be considered as well. The scattering of a plane wave from a cylinder is given analytically as

$$p^{\text{scat}}(r,\theta) = -p_0 \sum_{n=-\infty}^{\infty} i^n \frac{\frac{\mathrm{d}}{\mathrm{d}(ka)} J_n(ka)}{\frac{\mathrm{d}}{\mathrm{d}(ka)} H_n^{(1)}(ka)} H_n^{(1)}(kr) e^{in(\theta-\theta_0)} \tag{S55}$$

with $J_n(z)$ as a Bessel functions of n-th order, $H_n^{(1)}(z)$ as a Hankel function of the second kind of n-th order, a as the cylinder radius, θ_0 as the angle of incidence, k as the wave number, and r and θ as cylindrical coordinates. For small cylinders the scattered field can be described by monopole and dipole components corresponding to the $n = 0$ and $n = \pm 1$ terms. This simplifies the scattered field to

$$\begin{aligned} p^{\text{scat}}(r,\theta) = -p_0 \Bigg(& -i \frac{J'_{-1}(ka)}{H_{-1}^{(1)\prime}(ka)} H_{-1}^{(1)}(kr) e^{-i(\theta-\theta_0)} + \\ & + \frac{J'_0(ka)}{H_0^{(1)\prime}(ka)} H_0^{(1)}(kr) + \\ & + i \frac{J'_1(ka)}{H_1^{(1)\prime}(ka)} H_1^{(1)}(kr) e^{i(\theta-\theta_0)} \Bigg) \end{aligned} \tag{S56}$$

or

$$p^{\text{scat}}(r,\theta) = \gamma_{-1} H_{-1}^{(1)}(kr) e^{-i(\theta-\theta_0)} + \gamma_0 H_0^{(1)}(kr) + \gamma_1 H_1^{(1)}(kr) e^{i(\theta-\theta_0)}. \tag{S57}$$

Using Eq. (S14) the monopole moment can be calculated as

$$M = \frac{4}{ik^2c^2} \frac{J'_0(ka)}{H_0^{(1)\prime}(ka)} p_0 \tag{S58}$$

and after substituting $p_0 = p^{\text{inc}}$ for $x = 0$ and $\theta = 0$ the monopole component of the polarizability tensor appears as

$$\alpha_{pp}^{\text{cyl}} = \frac{4}{ik^2c^2} \frac{J'_0(ka)}{H_0^{(1)\prime}(ka)}. \tag{S59}$$

Evaluating the derivatives $J'_0(ka) = J_{-1}(ka) = -J_1(ka)$ and $H_0^{(1)\prime}(ka) = -H_1^{(1)}(ka)$ [3] leads to

$$\alpha_{pp}^{\text{cyl}} = \frac{4}{ik^2c^2} \frac{J_1(ka)}{H_1^{(1)}(ka)}. \tag{S60}$$

For the dipole moment we can perform an analogous process by substituting $\gamma_{-1,1}$ from (S56) into (S16). Due to symmetry, it suffices to consider only a single component of the dipole moment. We consider incident wave propagation parallel to the x-axis with $\theta_0 = 0$ so that $e^{i\theta_0} = 1$, yielding

$$D_x = \frac{4}{k^3c^2}\left(\frac{J_1'(ka)}{H_1^{(1)\prime}(ka)} + \frac{J_{-1}'(ka)}{H_{-1}^{(1)\prime}(ka)}\right)p_0, \tag{S61}$$

which can be further simplified to

$$D_x = \frac{8}{k^3c^2}\frac{J_1'(ka)}{H_1^{(1)\prime}(ka)}p_0. \tag{S62}$$

Substituting the expression $p_0 = \rho_0 c v_0$ results in the final equation

$$D_x = \frac{8\rho_0}{k^3c}\frac{J_1'(ka)}{H_1^{(1)\prime}(ka)}v_0, \tag{S63}$$

which gives us the polarizability tensor component

$$\alpha_{vv}^{\text{cyl}} = \frac{8\rho_0}{k^3c}\frac{J_1'(ka)}{H_1^{(1)\prime}(ka)}. \tag{S64}$$

Doe to the symmetry of a cylinder no Willis coupling is present and we can write the polarizability tensor as

$$\boldsymbol{\alpha}_{\text{cyl}} = \begin{bmatrix} \alpha_{pp}^{\text{cyl}} & 0 \\ 0 & \alpha_{vv}^{\text{cyl}} \end{bmatrix} = \begin{bmatrix} \frac{4}{ik^2c^2}\frac{J_1(ka)}{H_1^{(1)}(ka)} & 0 \\ 0 & \frac{8\rho_0}{k^3c}\frac{J_1'(ka)}{H_1^{(1)\prime}(ka)} \end{bmatrix} \tag{S65}$$

and its inverse as

$$\boldsymbol{\alpha}_{\text{cyl}}^{-1} = \begin{bmatrix} \frac{ik^2c^2}{4}\frac{H_1^{(1)}(ka)}{J_1(ka)} & 0 \\ 0 & \frac{k^3c}{8\rho_0}\frac{H_1^{(1)\prime}(ka)}{J_1'(ka)} \end{bmatrix}. \tag{S66}$$

Fig. 2(c, d) shows analytically (solid lines) and numerically determined (square and triangle markers) polarizability of a cylinder. Here the cylinder radius $a = 50\,\text{mm}$, $c = 343\,\text{m/s}$ and $\rho_0 = 1.2\,\text{kg/m}^3$. The analytical solution accurately matches the results extracted from the numerically calculated scattered field. The extracted off-diagonal components have a maximum dimension of 10^{-12}, which is equal to zero within numerical precision and hence the symmetry condition $\boldsymbol{\alpha}' = \boldsymbol{\alpha}'^{T-}$ [2] is trivially satisfied. This results validates the extraction method in combination with the custom BEM code.

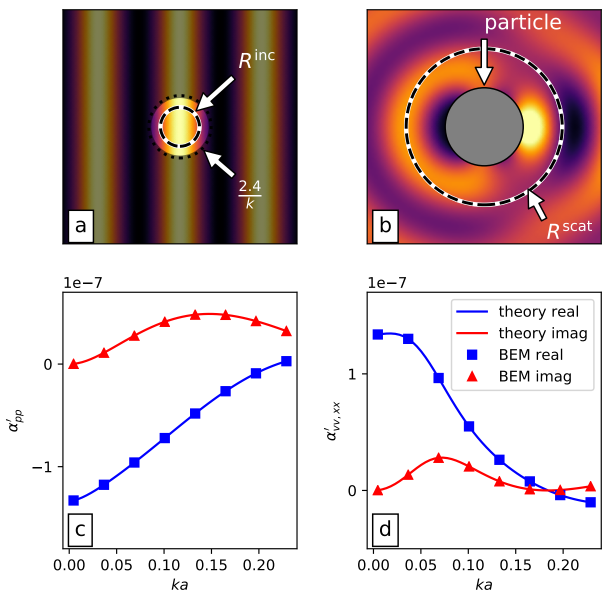

Supplementary Figure 2. Scattering from a cylinder. a: Illustration of chosen value of R^{inc}, for incident pressure field without particle. Dark region above $r = \frac{2.4}{k}$ can cause Eq. (S8) to become singular and should be avoided. b: Illustration of chosen value of R^{scat}, for the scattered pressure field from a cylinder. c: Monopole polarizability of a cylinder α'_{pp} given by theory (solid lines) and determined with BEM (square and triangle markers). d: Dipole polarizability of a cylinder α'_{vv}.

SUPPLEMENTARY NOTE 5:
RESONATOR-CYLINDER COUPLING

Analytical Coupling of Cylinder and Resonator in 1D

The meta-atom consists of a resonator inside a cylindrical body, and both objects contribute significantly to scattering. Furthermore the incident field for each object is influenced by the other, which implies that a coupled model is required.

General Coupling Model

The incident field of cylinder and resonator models can be expressed as

$$\begin{aligned}\breve{p}_{\text{inc}}^{\text{cyl}} &= \breve{p}_{\text{inc}} + \breve{p}_{\text{scat}}^{\text{res}} \\ \breve{p}_{\text{inc}}^{\text{res}} &= \breve{p}_{\text{inc}} + \breve{p}_{\text{scat}}^{\text{cyl}}\end{aligned} \tag{S67}$$

taking the influence by each other into account. Considering the cylinder the scattered field can be calculated as

$$\begin{bmatrix}\breve{p}\\ \breve{\mathbf{v}}\end{bmatrix}_{\text{scat}}^{\text{cyl}} = \underbrace{\begin{bmatrix}\frac{1}{2} & \frac{1}{2}\\ -\frac{1}{i2a\rho_0\omega} & \frac{1}{i2a\rho_0\omega}\end{bmatrix}\begin{bmatrix}f_M(r_1) & f_D(r_1,\theta_1)\\ f_M(r_2) & f_D(r_2,\theta_2)\end{bmatrix}}_{\mathbf{E}}\begin{bmatrix}M\\ \mathbf{D}\end{bmatrix}^{\text{cyl}} \tag{S68}$$

where $f_M(r)$ and $f_D(r,\theta)$ are the parts of (S13) and S15 corresponding to monopole and dipole moment as

$$f_M(r) = -\frac{ik^2c^2}{4}H_0^{(1)}(kr) \tag{S69}$$

and

$$f_D(r,\theta) = -\frac{ik^3c^2}{4}H_1^{(1)}(kr)\cos(\theta). \tag{S70}$$

For symmetrical aperture the coordinates of inlets are $r_1 = r_2 = a$ and $\theta_1 = \theta_2 + \pi$, which implies

$$\begin{aligned}f_M(r_1) = f_M(r_2) = f_M(a) &= f_M\\ -f_D(r_1,\theta_1) = f_D(r_2,\theta_1) = f_D(a) &= f_D\end{aligned} \tag{S71}$$

and

$$\mathbf{E} = \begin{bmatrix}f_M & 0\\ 0 & \frac{f_D}{ia\rho_0\omega}\end{bmatrix}. \tag{S72}$$

After substituting the moments with polarizability tensor $\boldsymbol{\alpha}$ and incident field $\mathbf{u}^{\mathrm{inc}} = \begin{bmatrix}\breve{p}^{\mathrm{inc}} & \breve{\mathbf{v}}^{\mathrm{inc}}\end{bmatrix}^T$ we end up with

$$\mathbf{u}_{\mathrm{scat}}^{\mathrm{cyl}} = \mathbf{E}\boldsymbol{\alpha}_{\mathrm{cyl}}\mathbf{u}_{\mathrm{inc}}^{\mathrm{cyl}} \tag{S73}$$

which incorporated into Eq. (S67) results in

$$(\mathbf{E}\boldsymbol{\alpha}_{\mathrm{cyl}})^{-1}\mathbf{u}_{\mathrm{scat}}^{\mathrm{cyl}} = \mathbf{u}_{\mathrm{inc}} + \mathbf{u}_{\mathrm{scat}}^{\mathrm{res}}. \tag{S74}$$

After completing the same consideration for the resonator we obtain the following two equations

$$\begin{aligned}\mathbf{u}_{\mathrm{inc}} &= (\mathbf{E}\boldsymbol{\alpha}_{\mathrm{cyl}})^{-1}\mathbf{u}_{\mathrm{scat}}^{\mathrm{cyl}} - \mathbf{u}_{\mathrm{scat}}^{\mathrm{res}} \\ \mathbf{u}_{\mathrm{inc}} &= (\mathbf{E}\boldsymbol{\alpha}_{\mathrm{res}})^{-1}\mathbf{u}_{\mathrm{scat}}^{\mathrm{res}} - \mathbf{u}_{\mathrm{scat}}^{\mathrm{cyl}}\end{aligned} \tag{S75}$$

which we combine to a system of equations

$$\begin{bmatrix}\mathbf{u}_{\mathrm{inc}} \\ \mathbf{u}_{\mathrm{inc}}\end{bmatrix} = \begin{bmatrix}(\mathbf{E}\boldsymbol{\alpha}_{\mathrm{cyl}})^{-1} & -\mathbf{I} \\ -\mathbf{I} & (\mathbf{E}\boldsymbol{\alpha}_{\mathrm{res}})^{-1}\end{bmatrix}\begin{bmatrix}\mathbf{u}_{\mathrm{scat}}^{\mathrm{cyl}} \\ \mathbf{u}_{\mathrm{scat}}^{\mathrm{res}}\end{bmatrix}. \tag{S76}$$

The scattered pressure can be calculated from the monopole and dipole moments (S13), (S15) and we get

$$\begin{bmatrix}\mathbf{u}_{\mathrm{inc}} \\ \mathbf{u}_{\mathrm{inc}}\end{bmatrix} = \begin{bmatrix}(\mathbf{E}\boldsymbol{\alpha}_{\mathrm{cyl}})^{-1} & -\mathbf{I} \\ -\mathbf{I} & (\mathbf{E}\boldsymbol{\alpha}_{\mathrm{res}})^{-1}\end{bmatrix}\begin{bmatrix}\mathbf{E} & \mathbf{0} \\ \mathbf{0} & \mathbf{E}\end{bmatrix}\begin{bmatrix}\begin{bmatrix}M & D\end{bmatrix}_{\mathrm{cyl}}^T \\ \begin{bmatrix}M & D\end{bmatrix}_{\mathrm{res}}^T\end{bmatrix} \tag{S77}$$

and after matrix multiplication we end up with

$$\begin{bmatrix}\breve{p}_{\mathrm{inc}} \\ \breve{v}_{\mathrm{inc}} \\ \breve{p}_{\mathrm{inc}} \\ \breve{v}_{\mathrm{inc}}\end{bmatrix} = \begin{bmatrix}\begin{bmatrix}\alpha_{pp} & \alpha_{pv} \\ \alpha_{vp} & \alpha_{vv}\end{bmatrix}_{\mathrm{cyl}}^{-1} & -\mathbf{E} \\ -\mathbf{E} & \begin{bmatrix}\alpha_{pp} & \alpha_{pv} \\ \alpha_{vp} & \alpha_{vv}\end{bmatrix}_{\mathrm{res}}^{-1}\end{bmatrix}\begin{bmatrix}M_{\mathrm{cyl}} \\ D_{\mathrm{cyl}} \\ M_{\mathrm{res}} \\ D_{\mathrm{res}}\end{bmatrix}. \tag{S78}$$

Coupling between Cylinder and Helmholtz Resonator in the Absence of Willis Coupling

When the structure has mirror symmetry, the Willis coupling terms α_{pv} and α_{vp} are zero for the Helmholtz resonator. The Willis coupling terms of the cylinder are always zero, so

we end up with a simplified system of equations

$$\begin{bmatrix} \breve{p}_{\text{inc}} \\ \breve{v}_{\text{inc}} \\ \breve{p}_{\text{inc}} \\ \breve{v}_{\text{inc}} \end{bmatrix} = \begin{bmatrix} (\alpha_{pp}^{\text{cyl}})^{-1} & 0 & -f_M & 0 \\ 0 & (\alpha_{vv}^{\text{cyl}})^{-1} & 0 & -\frac{f_D}{ia\rho_0\omega} \\ -f_M & 0 & (\alpha_{pp}^{\text{res}})^{-1} & 0 \\ 0 & -\frac{f_D}{ia\rho_0\omega} & 0 & (\alpha_{vv}^{\text{res}})^{-1} \end{bmatrix} \begin{bmatrix} M_{\text{cyl}} \\ D_{\text{cyl}} \\ M_{\text{res}} \\ D_{\text{res}} \end{bmatrix} = \mathbf{B}^{-1} \begin{bmatrix} M_{\text{cyl}} \\ D_{\text{cyl}} \\ M_{\text{res}} \\ D_{\text{res}} \end{bmatrix} \tag{S79}$$

where the matrix $\mathbf{B}$ is the total coupling matrix, which connect the incident field to the individual monopole and dipole moments. In absence of Willis coupling the monopole and dipole moments are completely uncoupled from each other and this allows to separate them into two independent matrix equations

$$\begin{bmatrix} \breve{p}_{\text{inc}} \\ \breve{p}_{\text{inc}} \end{bmatrix} = \begin{bmatrix} (\alpha_{pp}^{\text{cyl}})^{-1} & -f_M \\ -f_M & (\alpha_{pp}^{\text{res}})^{-1} \end{bmatrix} \begin{bmatrix} M_{\text{cyl}} \\ M_{\text{res}} \end{bmatrix} \tag{S80}$$

and

$$\begin{bmatrix} \breve{v}_{\text{inc}} \\ \breve{v}_{\text{inc}} \end{bmatrix} = \begin{bmatrix} (\alpha_{vv}^{\text{cyl}})^{-1} & -\frac{f_D}{ia\rho_0\omega} \\ -\frac{f_D}{ia\rho_0\omega} & (\alpha_{vv}^{\text{res}})^{-1} \end{bmatrix} \begin{bmatrix} D_{\text{cyl}} \\ D_{\text{res}} \end{bmatrix} \tag{S81}$$

The monopole moments can be now obtained by matrix inversion as

$$\begin{bmatrix} M_{\text{cyl}} \\ M_{\text{res}} \end{bmatrix} = \frac{1}{(\alpha_{pp}^{\text{cyl}})^{-1}(\alpha_{pp}^{\text{res}})^{-1} - f_M^2} \begin{bmatrix} (\alpha_{pp}^{\text{res}})^{-1} & f_M \\ f_M & (\alpha_{pp}^{\text{cyl}})^{-1} \end{bmatrix} \begin{bmatrix} \breve{p}_{\text{inc}} \\ \breve{p}_{\text{inc}} \end{bmatrix} \tag{S82}$$

and summed to give the total monopole moment

$$M_{\text{tot}} = M_{\text{cyl}} + M_{\text{res}}. \tag{S83}$$

The resulting equation

$$M_{\text{tot}} = \frac{(\alpha_{pp}^{\text{cyl}})^{-1} + (\alpha_{pp}^{\text{res}})^{-1} + 2f_M}{(\alpha_{pp}^{\text{cyl}})^{-1}(\alpha_{pp}^{\text{res}})^{-1} - f_M^2} \breve{p}_{\text{inc}} \tag{S84}$$

gives the uncoupled monopole component of the total polarizability tensor

$$\alpha_{pp}^{tot} = \frac{(\alpha_{pp}^{\text{cyl}})^{-1} + (\alpha_{pp}^{\text{res}})^{-1} + 2f_M}{(\alpha_{pp}^{\text{cyl}})^{-1}(\alpha_{pp}^{\text{res}})^{-1} - f_M^2}. \tag{S85}$$

The dipole moment can be determined in the same way from

$$\begin{bmatrix} D_{\text{cyl}} \\ D_{\text{res}} \end{bmatrix} = \frac{1}{(\alpha_{vv}^{\text{cyl}})^{-1}(\alpha_{vv}^{\text{res}})^{-1} - (\frac{f_D}{ia\rho_0\omega})^2} \begin{bmatrix} (\alpha_{vv}^{\text{res}})^{-1} & \frac{f_D}{ia\rho_0\omega} \\ \frac{f_D}{ia\rho_0\omega} & (\alpha_{vv}^{\text{res}})^{-1} \end{bmatrix} \begin{bmatrix} \breve{v}_{\text{inc}} \\ \breve{v}_{\text{inc}} \end{bmatrix} \tag{S86}$$

as a sum

$$D_{tot} = D_{\text{cyl}} + D_{\text{res}}. \tag{S87}$$

It gives us the total dipole moment as

$$D_{\text{tot}} = \frac{(\alpha_{vv}^{\text{cyl}})^{-1} + (\alpha_{vv}^{\text{res}})^{-1} + \frac{2f_D}{ia\rho_0\omega}}{(\alpha_{vv}^{\text{cyl}})^{-1}(\alpha_{vv}^{\text{res}})^{-1} - (\frac{f_D}{ia\rho_0\omega})^2}\breve{v}_{\text{inc}} \tag{S88}$$

and the corresponding component of the polarizability tensor

$$\alpha_{vv}^{tot} = \frac{(\alpha_{vv}^{\text{cyl}})^{-1} + (\alpha_{vv}^{\text{res}})^{-1} + \frac{2f_D}{ia\rho_0\omega}}{(\alpha_{vv}^{\text{cyl}})^{-1}(\alpha_{vv}^{\text{res}})^{-1} - (\frac{f_D}{ia\rho_0\omega})^2}. \tag{S89}$$

Finally the total polarizability tensor is defined as

$$\boldsymbol{\alpha}^{tot} = \begin{bmatrix} \alpha_{pp}^{tot} & 0 \\ 0 & \alpha_{vv}^{tot} \end{bmatrix}. \tag{S90}$$

Willis Coupling with two Apertures

To get the Willis coupling in the particle the symmetry of the resonator has to be disturbed. This can be easily achieved by changing the ratio of the aperture sizes to be unequal. This implies the appearence of Willis coupling term α_{pv} and α_{vp} in $\boldsymbol{\alpha}^{\text{res}}$ and the coupling matrix $\mathbf{B}^{-1}$ is written as

$$\begin{bmatrix} \breve{p}_{\text{inc}} \\ \breve{v}_{\text{inc}} \\ \breve{p}_{\text{inc}} \\ \breve{v}_{\text{inc}} \end{bmatrix} = \begin{bmatrix} (\alpha_{pp}^{\text{cyl}})^{-1} & 0 & -f_M & 0 \\ 0 & (\alpha_{vv}^{\text{cyl}})^{-1} & 0 & -\frac{f_D}{ia\rho_0\omega} \\ -f_M & 0 & (\alpha_{pp}^{\text{res}})^{-1} & (\alpha_{pv}^{\text{res}})^{-1} \\ 0 & -\frac{f_D}{ia\rho_0\omega} & (\alpha_{vp}^{\text{res}})^{-1} & (\alpha_{vv}^{\text{res}})^{-1} \end{bmatrix} \begin{bmatrix} M_{\text{cyl}} \\ D_{\text{cyl}} \\ M_{\text{res}} \\ D_{\text{res}} \end{bmatrix} = \mathbf{B}^{-1} \begin{bmatrix} M_{\text{cyl}} \\ D_{\text{cyl}} \\ M_{\text{res}} \\ D_{\text{res}} \end{bmatrix} \tag{S91}$$

Now the monopole and dipole moment equations can not be treated separately and the total matrix $\mathbf{B}^{-1}$ has to be inverted

$$\begin{bmatrix} M_{\text{cyl}} \\ D_{\text{cyl}} \\ M_{\text{res}} \\ D_{\text{res}} \end{bmatrix} = \mathbf{B} \begin{bmatrix} \breve{p}_{\text{inc}} \\ \breve{v}_{\text{inc}} \\ \breve{p}_{\text{inc}} \\ \breve{v}_{\text{inc}} \end{bmatrix} = \begin{bmatrix} \beta_{ij} \end{bmatrix} \begin{bmatrix} \breve{p}_{\text{inc}} \\ \breve{v}_{\text{inc}} \\ \breve{p}_{\text{inc}} \\ \breve{v}_{\text{inc}} \end{bmatrix}. \tag{S92}$$

From the uncoupled case we know that $M_{tot} = M_{\text{cyl}} + M_{\text{res}}$ and $D_{tot} = D_{\text{cyl}} + D_{\text{res}}$ and we can write the total moments as

$$\begin{bmatrix} M_{tot} \\ D_{tot} \end{bmatrix} = \begin{bmatrix} (\beta_{11} + \beta_{13} + \beta_{31} + \beta_{33}) & (\beta_{12} + \beta_{14} + \beta_{32} + \beta_{34}) \\ (\beta_{21} + \beta_{23} + \beta_{41} + \beta_{43}) & (\beta_{22} + \beta_{24} + \beta_{42} + \beta_{44}) \end{bmatrix} \begin{bmatrix} \breve{p}_{\text{inc}} \\ \breve{v}_{\text{inc}} \end{bmatrix} \tag{S93}$$

Finally the total polarizability tensor for presence of Willis coupling is obtained as

$$\boldsymbol{\alpha}^{tot} = \begin{bmatrix} (\beta_{11} + \beta_{13} + \beta_{31} + \beta_{33}) & (\beta_{12} + \beta_{14} + \beta_{32} + \beta_{34}) \\ (\beta_{21} + \beta_{23} + \beta_{41} + \beta_{43}) & (\beta_{22} + \beta_{24} + \beta_{42} + \beta_{44}) \end{bmatrix}. \tag{S94}$$

Willis Coupling with one Aperture

When the resonator has only one aperture, the polarizability tensor is simplified, but at the same time is becomes non-invertible as it has a zero determinant. In this case the inversion can be avoided by multiplication of the corresponding coupling matrix rows with $\boldsymbol{\alpha}_{\text{res}}$

$$\begin{bmatrix} \mathbf{u}_{\text{inc}} \\ \boldsymbol{\alpha}_{\text{res}} \mathbf{u}_{\text{inc}} \end{bmatrix} = \begin{bmatrix} \boldsymbol{\alpha}_{\text{cyl}}^{-1} & -\mathbf{E} \\ -\boldsymbol{\alpha}_{\text{res}} \mathbf{E} & \boldsymbol{\alpha}_{\text{res}} \boldsymbol{\alpha}_{\text{res}}^{-1} \end{bmatrix} \begin{bmatrix} \begin{bmatrix} M & D \end{bmatrix}_{\text{cyl}}^{T} \\ \begin{bmatrix} M & D \end{bmatrix}_{\text{res}}^{T} \end{bmatrix}. \tag{S95}$$

We can expand it as

$$\begin{bmatrix} \breve{p}_{\text{inc}} \\ \breve{v}_{\text{inc}} \\ \alpha_{pp}^{\text{res}} \breve{p}_{\text{inc}} + \alpha_{pv}^{\text{res}} \breve{v}_{\text{inc}} \\ \alpha_{vp}^{\text{res}} \breve{p}_{\text{inc}} + \alpha_{vv}^{\text{res}} \breve{v}_{\text{inc}} \end{bmatrix} = \begin{bmatrix} (\alpha_{pp}^{\text{cyl}})^{-1} & 0 & -f_M & 0 \\ 0 & (\alpha_{vv}^{\text{cyl}})^{-1} & 0 & -\frac{f_D}{ia\rho_0\omega} \\ -\alpha_{pp}^{\text{res}} f_M & -\frac{\alpha_{pv}^{\text{res}} f_D}{ia\rho_0\omega} & 1 & 0 \\ -\alpha_{vp}^{\text{res}} f_M & -\frac{\alpha_{vv}^{\text{res}} f_D}{ia\rho_0\omega} & 0 & 1 \end{bmatrix} \begin{bmatrix} M_{\text{cyl}} \\ D_{\text{cyl}} \\ M_{\text{res}} \\ D_{\text{res}} \end{bmatrix} = \mathbf{B}'^{-1} \begin{bmatrix} M_{\text{cyl}} \\ D_{\text{cyl}} \\ M_{\text{res}} \\ D_{\text{res}} \end{bmatrix} \tag{S96}$$

and analogously to the two aperture case form the total polarizability tensor

$$\boldsymbol{\alpha}^{tot} = \begin{bmatrix} \begin{pmatrix} \beta_{11} + \beta_{31} + \\ +\alpha_{pp}^{\text{res}} (\beta_{13} + \beta_{33}) + \\ +\alpha_{pv}^{\text{res}} (\beta_{14} + \beta_{34}) \end{pmatrix} & \begin{pmatrix} \beta_{12} + \beta_{32} + \\ +\alpha_{pv}^{\text{res}} (\beta_{13} + \beta_{33}) + \\ +\alpha_{vv}^{\text{res}} (\beta_{14} + \beta_{34}) \end{pmatrix} \\ \begin{pmatrix} \beta_{21} + \beta_{41} + \\ +\alpha_{pp}^{\text{res}} (\beta_{23} + \beta_{43}) + \\ +\alpha_{pv}^{\text{res}} (\beta_{13} + \beta_{33}) \end{pmatrix} & \begin{pmatrix} \beta_{22} + \beta_{42} + \\ +\alpha_{vp}^{\text{res}} (\beta_{23} + \beta_{43}) + \\ +\alpha_{vv}^{\text{res}} (\beta_{13} + \beta_{33}) \end{pmatrix} \end{bmatrix}. \tag{S97}$$

SUPPLEMENTARY NOTE 6:
EFFECTIVE LENGTH AND RADIATIVE LOSSES CORRECTION

The commonly used formulas for radiative damping and mass [3], are specific to circular geometries, and require modification for the case of our meta-atom. The radiative losses dictate the amplitudes of the polarizability tensor components and are of crucial importance for correctly determining Willis coupling coefficients. Furthermore the radiative mass is represented by adjusting the effective length of the apertures to correctly match the numerical results. This influences the resonant frequency.

For finetuning of these parameter we spanned a grid in parameter space over $w = [3, 12]$ mm and $l = [5, 10]$ mm where for every parameter combination the polarizability tensor was numerically determined. The numerical results were used for nonlinear optimization of the effective length correction coefficient c_{eff} and radiation loss coefficient c_{rad}. These coefficients are incorporated into the model through the equations

$$l_{\mathrm{eff}} = l + c_{\mathrm{eff}} \tag{S98}$$

and

$$K_{\mathrm{eq}} = i\omega^3 c_{\mathrm{rad}} \frac{\rho_0 A}{c} + \omega^2 \rho_0 l_{\mathrm{eff}} - \frac{K}{V} A, \tag{S99}$$

which implies that

$$\boldsymbol{\alpha} = f\left(c_{\mathrm{eff}}, c_{\mathrm{rad}}, \omega ...\right). \tag{S100}$$

The optimization function is based on the Willis coupling component α_{pv} and is defined as

$$f_{opt}\left(c_{\mathrm{eff}}, c_{\mathrm{rad}}\right) = \|\alpha_{pv}^{BEM}\left(\omega\right) - \alpha_{pv}^{analyt}\left(\omega, c_{\mathrm{eff}}, c_{\mathrm{rad}}\right)\|_2 \longrightarrow \min. \tag{S101}$$

Minimizing this function for every point in (l, w) space gives us a c_{eff}-surface and a c_{rad}-surface. These surfaces represent the optimal values for the evaluated points, which can be now approximated with simple terms in l, w-space. This terms were found by the means of linear regression and the resulting function are

$$c_{\mathrm{eff}}\left(l, w\right) \approx w\left(C_0^{\mathrm{eff}} + C_1^{\mathrm{eff}} \cdot w + C_2^{\mathrm{eff}} \cdot w^2 + C_3^{\mathrm{eff}} \cdot w \cdot l\right) \tag{S102}$$

with coefficients

$$
\begin{aligned}
C_0^{\text{eff}} &= 2.36 \\
C_1^{\text{eff}} &= -1.58 \times 10^2\,\text{m}^{-1} \\
C_2^{\text{eff}} &= 4.71 \times 10^3\,\text{m}^{-2} \\
C_3^{\text{eff}} &= 8.47 \times 10^2\,\text{m}^{-2}
\end{aligned}
\tag{S103}
$$

and

$$
c_{\text{rad}}\,(l, w) \approx C_0^{\text{rad}} + \frac{C_1^{\text{rad}}}{\sqrt{w}} + C_2^{\text{rad}} \cdot l + \frac{C_3^{\text{rad}}}{w \cdot l} \tag{S104}
$$

with coefficients

$$
\begin{aligned}
C_0^{\text{rad}} &= 9.3 \times 10^{-3} \\
C_1^{\text{rad}} &= 2.32 \times^{-4}\ \sqrt{\text{m}} \\
C_2^{\text{rad}} &= -2.7 \times^{-1}\ \text{m}^{-1} \\
C_3^{\text{rad}} &= 2.61 \times^{-8}\ \text{m}^2
\end{aligned}
\tag{S105}
$$

These equations were optimized to have a minimal number of terms and to produce a maximum error of less than 3%. A comparison between the fitted functions with the numerically calculated results is shown in Fig. 3. These expressions are substituted into Eqs. (S98) and (S99) for calculation of the polarizability tensor for the resonator with one aperture.

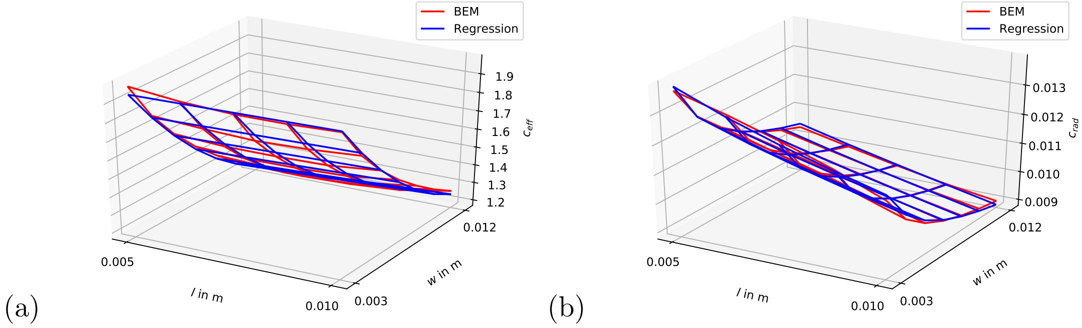

Supplementary Figure 3. Results of nonlinear optimization and subsequent linear regression for c_{eff} (a) and c_{rad} (b) in l, w-space.

SUPPLEMENTARY NOTE 7:
TAILORING THE WILLIS COUPLING BY VARYING META-ATOM GEOMETRY

Here we demonstrate how Willis coupling can be tailored by controlling the geometric parameters of the meta-atom. First, a single aperture meta-atom with $\frac{w_1}{w_2} = \infty$ is considered, similar to the experimentally investigated sample. Fig. 4(a-d) illustrates the normalized polarizability components α'_{pp}, α'_{pv}, α'_{vp}, and α'_{vv} determined analytically (blue, red and black lines) and numerically (green lines with markers). Theoretical and numerical polarizabilities are in perfect agreement. The maximum magnitude of Willis coupling is reached, as shown by α'_{pv} and α'_{vp} touching the theoretical bound (magenta line) in Fig. 4(b,c).

Next, a meta-atom with the same parameters but an additional smaller aperture is considered ($\frac{w_1}{w_2} = 4$). The resulting polarizability is shown in Fig. 4(e-h), where theory perfectly matches the BEM results. Two important effects are observable. First, the eigenfrequency (and therefore the frequency of peak Willis coupling) is shifted, which can be understood through Eq. (S40). Second, the Willis coupling amplitude is reduced, see Fig. 4(f,g), since the asymmetry of the particle is weaker. Furthermore, the profile of α'_{pp} in Fig. 4(e) shows no significant difference from Fig. 4(a). In contrast, the profile of α'_{vv} is strongly reduced in amplitude close to the resonator eigenfrequency. The effect of decreased Willis coupling (see Fig. 4(f,g)) is of practical importance, since it can be used to tailor the Willis coupling to required values.

A special case is the symmetrical double aperture meta-atom, where $\frac{w_1}{w_2} = 1$. Its polarizability given in Fig. 4(i-l) differs significantly from the asymmetrical meta-atoms discussed above. The resonance frequency is further shifted due to the change of the resonator parameters (see Eq. (S40)). The Willis coupling is zero, which agrees with the expectations due to the symmetry of the structure. The α_{pp} component in Fig. 4(i) shows no significant change compared to previous parameter choices. Interestingly, α_{vv} no longer has a peak and moreover, it approaches the polarizability of a cylinder given by Eq. (S66). In this case two design points could be of interest, one in the peak of α_{pp} by $ka = 1.15$ and one in the zero dip of it by $ka = 0.75$ (see Fig. 4(i)). Especially in the zero dip, a regime with a dipole response of a cylinder, but without any or with extremely weak monopole response is obtained.

The ratio $\frac{w_1}{w_2}$ allows Willis coupling to be engineered, however changing the ratio of widths also shifts the peak frequency, as can be observed in Fig. 5. To obtain a meta-atom of the

same size and with constant peak frequency varying only Willis coupling requires adjustment of additional parameters. This relation can be observed in Fig. 6, which considers neck widths in the range $0.15 \leq \frac{w}{a} \leq 0.6$. This range is limited to avoid very narrow slits which would lead to high thermo-viscous losses. The colored background refers to peak Willis coupling normalized to the theoretical bound and the contours illustrate the peak frequencies. Taking for example a meta-atom of size $a = 20\,\text{mm}$: engineering of different Willis coupling peak magnitudes at $ka = 0.9$ equates to choosing w_1 and w_2 from Fig. 6 along the dashed teal like corresponding to $ka = 0.9$.

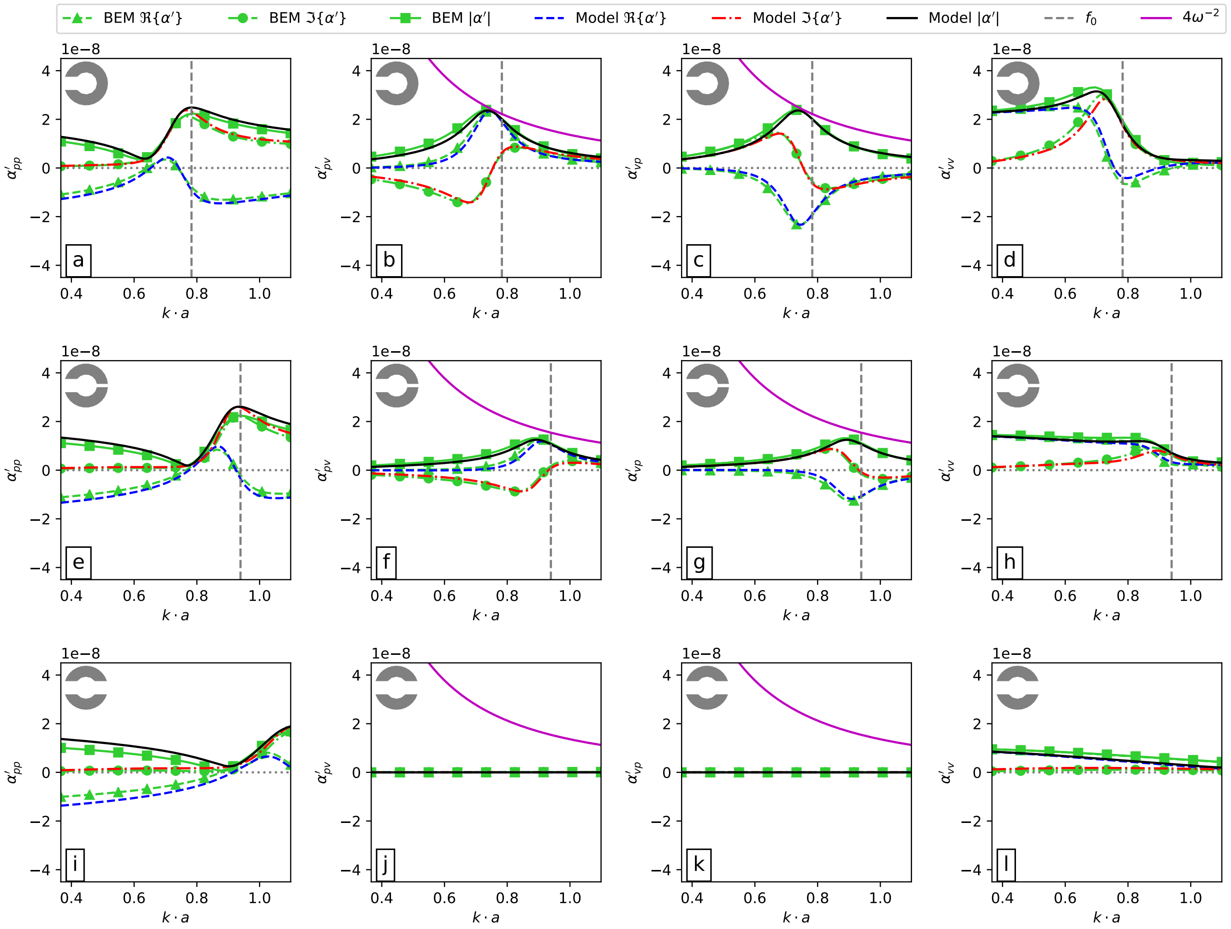

Supplementary Figure 4. Meta-atom polarizability determined by theory (blue, red and black lines) and numerically (green lines with markers): a, b, c, d: Single aperture meta-atom with $a = 20$ mm, $l = 10$ mm and $w = 12$ mm. e, f, g, h: Double aperture meta-atom with $a = 20$ mm, $l = 10$ mm and $w_1 = 12$ mm and $w_2 = 3$ mm. i, j, k, l: Symmetrical double aperture meta-atom with $a = 20$ mm, $l = 10$ mm and $w_1 = 3$ mm and $w_2 = 3$ mm.

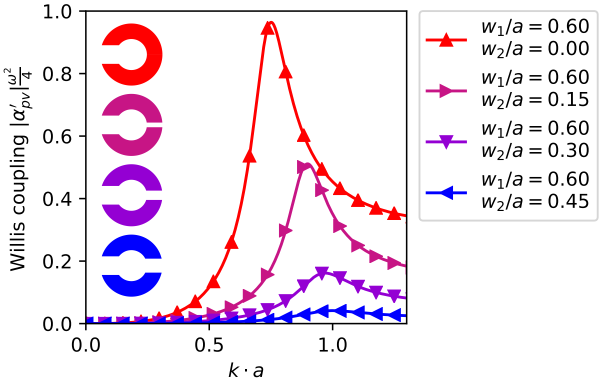

Supplementary Figure 5. Willis coupling of four different meta-atom geometries through variation of w_2. Peak Willis coupling and peak frequency are changed.

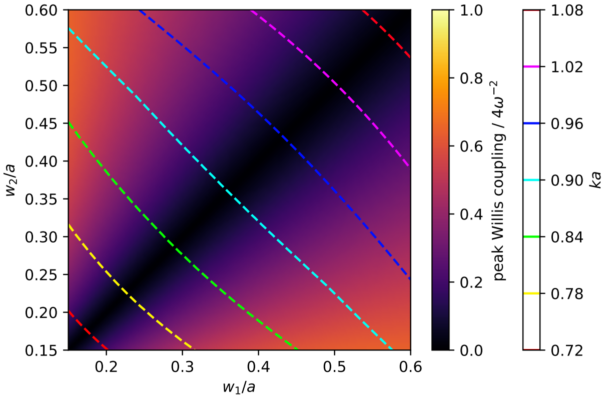

Supplementary Figure 6. Design space of double aperture meta-atom: Willis coupling normalized to the maximum bound as colored background and contour lines of peak frequencies as colored dashed lines.

SUPPLEMENTARY REFERENCES

1. Jordaan, J. *et al.* Measuring Monopole and Dipole Polarizability of Acoustic Meta-Atoms. *Applied Physics Letters* **113,** 224102. ISSN: 0003-6951 (Nov. 2018).

2. Quan, L., Ra'di, Y., Sounas, D. L. & Alù, A. Maximum Willis Coupling in Acoustic Scatterers. *Physical Review Letters* **120.** ISSN: 0031-9007, 1079-7114. doi:10.1103/PhysRevLett.120.254301. https://link.aps.org/doi/10.1103/PhysRevLett.120.254301 (2018) (June 20, 2018).

3. Kinsler, L. E. *Fundamentals of acoustics* 568 pp. ISBN: 978-0-471-84789-2 (Wiley, 2000).

4. Crow, B. C., Cullen, J. M., McKenzie, W. W., Koju, V. & Robertson, W. M. Experimental realization of extraordinary acoustic transmission using Helmholtz resonators. *AIP Advances* **5,** 027114 (Feb. 1, 2015).

5. Dosch, H. G. Radiative feedback in Helmholtz resonators with more than one opening. *The Journal of the Acoustical Society of America* **140,** 3576–3581. ISSN: 0001-4966 (Nov. 1, 2016).
